# P-1947. Risk of Severe COVID-19 Reinfection among Persons Hospitalized with a Primary COVID-19 Infection, Minneapolis-St. Paul, Minnesota

**DOI:** 10.1093/ofid/ofae631.2106

**Published:** 2025-01-29

**Authors:** Haley Wienkes, Paige D’Heilly, Erica Bye, Stephanie Meyer, Kathryn Como-Sabetti, Ruth Lynfield

**Affiliations:** Minnesota Department of Health, St. Paul, Minnesota; Minnesota Department of Health, St. Paul, Minnesota; Minnesota Department of Health, St. Paul, Minnesota; Minnesota Department of Health, St. Paul, Minnesota; Minnesota Department of Health, St. Paul, Minnesota; Minnesota Department of Health, St. Paul, Minnesota

## Abstract

**Background:**

SARS CoV-2 continues to circulate broadly making it important to understand risk factors for severe illness when reinfection occurs. We used CDC COVID-19 Hospitalization Surveillance Network (COVID-NET) data to assess reinfection, hospitalization at reinfection (hospitalized reinfection) and ICU admission at reinfection (ICU reinfection) by underlying medical conditions and ICU admission at primary infection.

**Methods:**

Nonpregnant adults hospitalized 3/9/2020 - 9/30/2021 were identified through COVID-NET, surveillance that includes Minneapolis-St. Paul metropolitan area residents with a positive SARS-CoV-2 result during or ≤ 14 days before admission. Cases were matched to all reports of persons positive for SARS-CoV-2 to identify reinfections, defined as a positive SARS-CoV-2 test > 90 days after the initial positive result through 4/17/2024. Logistic regression was used to assess reinfection and severity of reinfection adjusted for age, sex, race/ethnicity, and vaccination status (aOR).

**Results:**

6,434 hospitalized persons met the COVID-NET case definition for their primary infection; 1,189 (18%) had a reinfection. 356 (30%) of the reinfection cases were hospitalized and 51 (14%) of those hospitalized had ICU admission. Having any underlying condition was associated with reinfection (aOR 1.62, 95% CI 1.18, 2.23) and hospitalized reinfection (aOR 2.16, 95% CI 1.27, 3.69). Cardiovascular disease was associated with reinfection (aOR 1.72, 95% CI 1.28, 2.31). Cardiovascular disease (aOR 3.03, 95% CI 1.87, 4.90), blood disorders (aOR 2.42, 95% CI 1.03, 5.37), and renal disease (aOR 1.85, 95% CI 1.12, 3.06) were associated with hospitalized reinfection. All 51 ICU reinfections and 14 reinfection deaths had an underlying condition. Primary ICU admission was not associated with reinfection or hospitalized reinfection but was associated with ICU reinfection (aOR 5.26, 95% CI 1.65, 16.73).

**Conclusion:**

Among patients hospitalized with COVID-19, underlying conditions and primary ICU admission were associated with severe outcomes during reinfection. These results highlight the continued importance of prevention strategies like vaccination and treatment to mitigate severe illness among patients at highest risk.

**Disclosures:**

All Authors: No reported disclosures

